# Quantitative Flow Cytometry—Medical Applications with a Focus on Blood Platelets

**DOI:** 10.3390/ijms27041976

**Published:** 2026-02-19

**Authors:** Philippe Poncelet, Thomas Lecompte, Anne Bauters, François Mullier

**Affiliations:** 1 R and T Department, BioCytex, 13010 Marseille, France; philippe.poncelet@stago.com; 2 Faculté de Médecine, Université de Lorraine, 54000 Nancy, France; thomas.lecompte@icloud.com; 3 Centre Hospitalier Universitaire de Lille (CHU Lille), 59000 Lille, France; anne.bauters@chu-lille.fr; 4 Department of Laboratory Medicine, CHU UCL Namur, Université Catholique de Louvain, 5530 Yvoir, Belgium

**Keywords:** quantitative flow cytometry, surface antigens, platelets

## Abstract

Flow cytometry, measuring light signals, can be used as a quantitative tool to appreciate the numbers of cell-surface molecules targeted with monoclonal antibodies, among other applications. This has been extensively used for blood cells and especially platelets. This review describes how techniques have evolved over time since the first developments of quantitative flow cytometry at the end of the 20th century. Technological issues are first described, applicable to all types of cells/molecules and largely relying on calibration beads with direct or, preferably, indirect immunofluorescence. The platelet field is then addressed with specific tools devoted to surface antigen quantitation. The array of commercially available kits is provided with their specificity. A panorama of platelet antigens quantified that can be used in the diagnosis workout of platelet disorders is then provided, accompanied by a reminder of the impressive stability of marker expression in normal individuals. Variations are then considered in the light of aging or genetic polymorphisms. Finally, the upcoming use of platelet antigen quantification as a monitoring tool for emerging targeted therapies is evoked. All in all, this review provides a comprehensive story of the evolution of the still too marginally used cell antigen quantification.

## 1. Introduction

The intrinsic properties of flow cytometry (FCM), measuring fluorescence intensities accurately, make it a valuable tool to appreciate disease-related variations in the expression of cell-surface antigens in the common case of using fluorochrome-labelled antibodies. The concept of “quantitative immunophenotype”, i.e., evaluating individual fluorescences with arbitrary units, has been investigated for many years and reviewed in a number of publications [1,2]. Indeed, quantitative FCM (QFCM) can provide valuable information in the follow-up of individual patients or groups of patients. However, perhaps because of some hindrances in proper standardization, this approach, which emerged at the end of the 20th century, does not appear to have gained the interest expected from its high potential. The application of FCM-based immunofluorescence (IF) analysis for in vitro diagnostics and patient management indeed requires proper standardization between instruments or the dedicated use of a single one [3,4,5]. The progress of instrumentation has provided great help, but this goal is still not met in all instances. With proper standardization, or rather harmonization [6], QFCM can be proposed as a valuable diagnostic tool in different settings, especially if normal ranges can be established and universally applied. Moreover, for rare conditions, this would involve multicenter studies, helpful to better understand the mechanisms of several diseases. Of note is the fact that QFCM, not being a functional test *per se*, can however reflect some biological functions, differentiation stages and/or activation status. It can also provide valuable information for patients receiving drugs targeting cell-surface antigens, notably in terms of monitoring. This review aims at highlighting the interest of QFCM in the realm of platelet disorders and involved mechanisms, first focusing on the technology applied and then providing information on different targets and applications as they developed over time.

## 2. Technology

### 2.1. Principle

The aim of QFCM [7] is to appreciate the quantity (number) of a given molecule expressed by specific cell subsets through the use of monoclonal antibodies (mAbs) recognizing an epitope on the molecule targeted, usually a differentiation antigen (Ag). The mean number of mAb molecules bound per cell, or antibody-binding capacity (ABC), can be measured under various stoichiometric conditions [8]. Ensuring saturating conditions provides the maximal reachable value of ABC. The latter is usually inferred to reflect the expression level of the target Ag (Ag molecules/cell), assuming that one mAb will bind to one Ag molecule (monovalent binding). This is most often the case under saturating conditions associated with mAb excess, using Ab (usually IgG) molecule numbers 100 or 1000 times higher than that of Ags. However, the literature provides a few examples where divalent binding is considered to be the prevalent situation. Since each mAb IgG carries two antibody-binding sites (Fab), it may happen that the same mAb molecule binds common epitopes of two close Ags (<50 Å), owing to the presence of Ag clusters. This happens, for instance, in the case of CD4 on lymphocytes [9], CD41 or CD61 on platelets [10], CD9-bound molecular complexes in membrane rafts [11] or GPVI dimers [12].

QFCM can be performed relatively easily, provided that enough care is given to staining protocols and that appropriate known levels of reference materials are included in the assay for calibration purposes. Various categories of calibrators, called “Multi-level, Type III, fluorescence Standards”, have been introduced during the 80s and 90s to “bring the Metry into Flow Cytometry”, as qualified by Schwartz et al. in 1998 [13].

### 2.2. Calibrators

As shown in Table 1, there are several types of calibrators, mostly represented by synthetic beads with a size enabling their easy gating in FCM.

The calibrators mentioned on the first row of Table 1 are known as “hard-dyed”. These beads are mainly devoted to instrument quality control, which includes verification of the sensitivity and performance of fluorescence detectors, Photo Multiplier Tubes (PMTs) and avalanche photodiodes. Regular use of these beads ensures instrument consistency over time, since they allow for the detection of changes in performance that may impact data. The ideal product, for this application, would feature dyes that are thermally and photolytically stable in the long term. These hard-dyed polymer beads are produced by a physicochemical process that incorporates the dye within the polymer matrix. These beads are generally stable for at least two years. The dyes in such hard-dyed beads are, however, not spectrally equivalent to the common dyes used in flow cytometry and should not be used to obtain absolute quantitation of fluorophores on cells, although some attempts tried to provide them with equivalent relative fluorescence units via re-calibration [14].

Indeed, fluorescence quantitation to determine the amount of fluorophore-conjugated mAb bound to the cell requires spectrally equivalent standard beads stained with the same dyes as those used to label the cells, as stated by Wang et al. [15]. These surface dyes of Type IIIB calibrators, as shown in the second row of Table 1, simulate different levels of dye that could be attached to stained cell membranes. Users are then able to estimate the number of dye molecules bound per cell by comparing cell fluorescence to that of a bead of known Molecules of Equivalent Soluble Fluorochrome (MESF) or Mean Equivalent Fluorochrome (MEFL). As a practical drawback, surface-stained beads in suspension are less stable than hard-dyed beads and, hence, have limited shelf life. Freeze-dried surface-stained beads, such as QuantiBrite^®^ [16], have a longer, although still limited, shelf life.

If the background noise remains significant (e.g., >5–10%) compared to the positive ABC signal, it is recommended to calculate a specific ABC (sABC) after correction by the apparent ABC of a concentration-matched isotypic control. This correction is tricky to apply in direct IF, due to a usually different fluorochrome-to-protein (F:P) ratio between mAb conjugates and their commercial isotypic controls. The alternative provided by indirect labeling, which uses the same conjugate for the specific and isotypic control mAbs, is a significant advantage. This is the case for the Quantitative ImmunoFluorescence Indirect (QIFI) assay and all derived specialized kits [17]. They enable robust measurements at density levels of a few thousand and even sometimes even a few hundred molecules per cell, as may occur for platelet activation Ags such as P-Selectin (CD62P), formerly known as GMP-140 or PADGEM (platelet-activation-dependent granule-external membrane protein) [18].

The difficulty of this apparently straightforward approach lies in the translation between MESF or MEFL in a number of mAb molecules. This calculation step requires knowledge about the F:P ratio of each conjugate used, mandatory information that is difficult to obtain, as it is generally not provided by the manufacturers and distributors of mAb conjugates. One specific exception is phycoerythrin (PE) conjugates, purposely made upon special request by BD Biosciences^®^ at a guaranteed F:P ratio of 1, for use with their PE-calibrated QuantiBrite^®^ beads [9]. Although Quantum Simply Cellular^®^ (QSC) beads (with a single high-level ABC) were proposed as a tool to determine F:P ratios when used in combination with fluorescent Quantum^®^ beads, these suffered from limitations in the reliability of their ABC.

Alternative systems aimed at measuring cell-bound mAb make use of intrinsically non-fluorescent beads (Type IIIC or D), as described in rows 3 and 4 of Table 1. They theoretically mimic cells in IF approaches. The design of QSC^®^ beads (Type IIIC, row 3) is to coat them with anti-mouse IgG (or alternatively anti-human or rat IgG) that will recognize the Fc part of conjugated mAbs, mimicking cell staining obtained by fluorescent mAb binding. By using different concentrations of coating, they provide multiple ABC levels to be compared with stained cells in direct IF assays, through calibration curves for each single Ab conjugate, with any kind of attached fluorescent molecule [8].

QIFIkit^®^ beads (Type IIID, row 4 of Table 1) were designed to mimic cells stained in indirect fluorescence. They are coated with mouse IgG and are recognized through the use of fluorochrome-conjugated anti-mouse IgG. Based on the demonstrated equivalent staining of all mouse IgG isotypes [19,20], this tool provides a single calibration curve that can be used for any mouse mAb, as illustrated during the clusters of differentiation classification workshops [21].

Derived from this concept and adapted to no-wash indirect IF staining protocols (Type IIID, row 4 of Table 1), CellQuant^®^ Calibrator beads (10 µm diameter, featuring bound mouse, rat or human IgG depending on the model) and Platelet Calibrator^®^ beads (3 µm diameter, covered with mouse IgG) are provided in suspension in a diluent containing mouse IgG at a concentration of 5 µg/mL and an appropriately titrated fluorescent secondary anti-IgG reagent, which ensures saturation staining without washing off the first mAb labeling layer. This is particularly useful when dealing with such sensitive cells as platelets, which may suffer from centrifugation-based washing steps, either by forming clumps or by being activated. Platelet-oriented kits include beads with a size close to that of resting platelets (3 µm), which have a discoid shape, with a diameter of 2–4 µm and a thickness of 0.5 µm, and sphericize upon activation. These beads have a maximal ABC (~100,000 molecules/bead) compatible with the maximal Ag densities observed on platelets.

Because it had been designed for use with current direct IF immunophenotyping protocols, the QSC system was considered, for several years, as the simplest and easiest way to achieve ABC calibration, until critical studies made it clear that quite different ABC values were measured with different conjugates of the same mAb against the same Ag [13,22,23]. ABC data thus were shown to fit well only with the QuantiBrite^®^ and QIFIkit^®^ systems and not with the QSC system [24]. These puzzling data could be explained by the different kinetics of mAb cell binding compared to bead binding [25]. Indeed, the QSC system relies on the recognition of the mAb by the coated anti-IgG, which binds the mAb Fc portion, and it depends on the affinity of the anti-IgG for the mAb molecule. Conversely, the mAb molecule recognizes a specific antigen on the cell surface, with its own, and likely different, affinity [26]. Such stoichiometric variations are more likely than differences in binding site saturation, which was another hypothesis where insufficient saturation on reference beads would lead to overestimation of the number of binding sites on the target cells, as demonstrated by Bikoué et al. [25]. This is illustrated by the much higher receptor densities reported by these authors as compared to previous published values [17].

Finally, Table 1 suggests that stabilized cells with known ABC values may also be considered to help towards absolute Ag quantitation. Although, in specific cases, they may enable calibration, as suggested by Bikoué et al. [25], their best value is to serve as controls with pre-established ABC values in QFCM protocols.

## 3. Specific Tools for Platelet Receptor Quantitation

**Figure 1:**
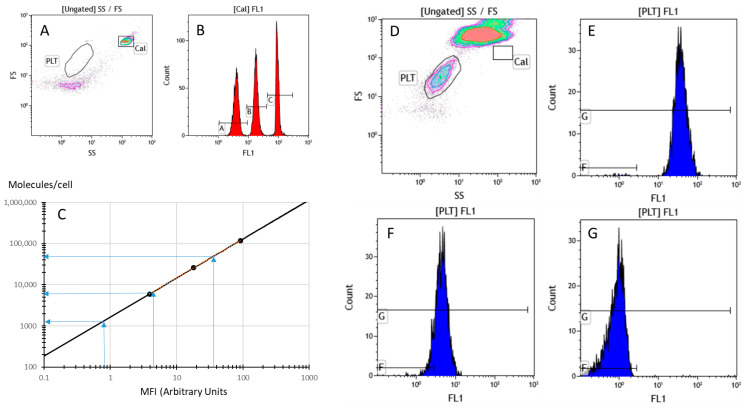
Indirect no-wash quantitation of platelet surface expression of CD41 and CD49b. (**A**) Gating of calibration beads (Cal); (**B**) monoparametric representation of the fluorescence of the three types of beads; (**C**) calibration curve derived from the fluorescences of beads; (**D**) whole-blood sample with platelet gating (PLT); (**E**) platelets stained with anti-CD41 mAb then FITC-conjugated anti-mouse IgG antibody; (**F**) platelets stained with anti-CD49b mAb then FITC-conjugated anti-mouse IgG antibody; (**G**) control of unstained platelets stained with irrelevant IgG, and FITC-conjugated anti-mouse IgG antibody.

Figure 1 illustrates the practical use of Platelet Calibrator^®^, a commercially available kit devoted to the quantitation of various platelet surface Ag recognized by mouse IgG mAbs.

Many mAbs can be used provided that they can saturate their target Ag at a final concentration of 5 µg/mL maximum, the optimized working amount in this “no-wash” IF indirect assay. Many examples of applications can be found in the literature, including measurements of specific and non-specific platelet glycoproteins (GPs), as shown in Table 2.

Briefly [27], whole blood, preferably collected in citrate-containing tubes to preserve platelets, or diluted platelet-rich plasma, is incubated for 10 min at room temperature with one or several mAbs, including an isotypic control, with one mAb per tube. Calibrator beads as well as mAb-saturated blood samples are then supplemented by the secondary fluorochrome-labeled (anti-mouse usually) Ab for an additional 10 min incubation, followed by a final dilution in phosphate-buffered saline (typically 1:50) and FCM analysis. The calibration curve provided by the beads is used for the calculation of ABC values (in mAb molecules/cell) from the MFI of each staining. Non-specific mAb binding is provided by the matched isotypic control and is subtracted to get the specific ABC (sABC), which corresponds to the Ag expression level.

Most often, only limited differences appear when quantitating the same target Ag using different mAbs, even with different isotypes, as illustrated by Poncelet et al. [19], Antal-Szalmas et al. [20] and Dupont et al. [27]. However, it may happen that different mAbs recognize different domains of the same membrane Ag with different accessibility or repeated epitopes. Another reason for differences can be insufficient affinity to guarantee saturation. This has been reported, for instance, for cellular prion protein on red blood cells [28]. In extreme cases, binding affinity can be different between mAbs depending on genetic polymorphisms. For this reason, pre-selected mAbs checked for Ag saturation are provided in the various Biocytex Platelet application kits^®^ (research use only [RUO]), so that reproducible measurements can be operated for the proposed targets such as CD41, CD42b and CD62P in the “PLT Gp/Receptors”^®^ kit (RUO).

Of note is the fact that this technology can also apply to subcellular fragments serving as vectors of inter-cellular communications. Extra-cellular vesicles (EVs) are either excreted from intra-cellular pools (exosomes) or formed by membrane budding (ectosomes or microvesicles, MVs). In addition to major efforts for the standardization of scatter-based analysis protocols [29,30,31], calibration is nowadays also requested to homogenize immunophenotypic studies of EVs. With this objective in mind, calibrators devoted to extending the applications of QFCM to EVs and help design vectorized therapeutic nanoparticles such as immuno-liposomes have been proposed [32].

## 4. Main Platelet Receptors Quantified

The kit that enables a precise quantitation of GPIIb (CD41), GPIb (CD42b) and P-Selectin (CD62P) on platelets, both at the resting state and after TRAP (Thrombin Receptor Agonist Peptide) activation, helps in detecting Inherited Platelet Disorders (IPDs) involving quantitative defects of the main platelet surface GPs, i.e., GPIIb/IIIa and GPIb/IX/V complexes, Glanzmann thrombasthenia (GT) [33] or biallelic or monoallelic Bernard–Soulier syndrome (BSS) [34,35] respectively, as well as platelet release defects [36]. CD62P becomes detectable after activation and exocytosis of alpha-granules, whereas the two main surface GP complexes increase and decrease, respectively (Table 3).

Quantification of platelet GPs for the diagnosis of IPDs is well mentioned in guidelines for patients with a bleeding disorder [37,38,39,40]. In biallelic BSS [36], a defect of the GPIbIX complex is observed in flow cytometry, whereas in monoallelic forms, only partial loss or normal GPIbIX complex density is observed.

Table 3 illustrates typical results obtained when the major platelet GPs are measured on resting or activated platelets collected twice at various dates from a healthy donor, from a patient with gray platelet syndrome (GPS), a primary IPD with a defect in alpha granules [41], and from a patient with biallelic BSS. Data is provided as sABC and can be compared between samples. The stability of measured densities is highlighted, strengthening the concept of a biological standard for expression levels of cell-surface Ags.

Other features coming out from the QFCM approach include (i) slightly higher amounts of GPIIIa (CD61) than the GPIIb/IIIa (CD41/CD61) complex, since GPIIIa, also known as the β3 component of integrins, is also part of the vitronectin receptor, (ii) the expected lower density of GPIb (CD42b) compared to GPIIb/IIIa observed on normal platelets but not on those of patients due to a lower-than-normal level of CD41, (iii) the expected increased levels of GPIIb/IIIa upon TRAP activation, (iv) the expected decreased levels of GPIb [42] upon TRAP activation, again more clearly on normal than on diseased platelets, (v) the expected membrane externalization of P-Selectin after TRAP activation on normal platelets and (vi) the explainable disease-related low level of externalized P-Selectin on abnormal platelets implying a defect of the α-granules not restricted to their content.

Finally, the interesting point is that such (old) [43] data are still up to date nowadays after more than 25 years, since human normal platelets have consistently been shown to bear about 50,000 GPIIb/IIIa ABC.

Normal values are especially valuable to readily interpret abnormal values encountered in pathological situations. Table 4 illustrates a family study where both parents were heterozygous for GT and a daughter was homozygous with only about one-third the normal amount of GPIIb/IIIa. Interestingly, the low level of GPIIb/IIIa on the daughter’s quiescent platelets was confirmed after TRAP activation, whereas the partial amounts observed on the parents’ platelets were almost doubled upon activation, confirming a lack of membrane expression of internal GPIIb/IIIa.

Finally, inherited *GFI1B* and *RUNX1* sequence variations, which cause inherited thrombocytopenia, are associated with abnormal platelet surface CD34 expression [44], normally restricted to hematopoietic stem cells and progenitors. Platelet α- and δ-granule numbers are often less abundant compared to normal platelets (CD62P and CD63 expression after activation, respectively).

Clinical platelet and molecular characteristics have been recently studied [45] in 37 patients with *RUNX1*-related thrombocytopenia (RT), nine with *ETV6*-RT and 20 with *ANRKD26*-RT, who are at high risk (10–45%) of developing hematological malignancy. Most *RUNX1*-RT patients had low GPIa levels, which may be a useful disease biomarker.

Of note is the fact that FCM is particularly suitable when small amounts of blood are available (neonates, infants) and/or when platelet counts are low (BSS, GPS) [43].

## 5. Normal Values and Their Modulation

In many examples, cell-surface Ag expression levels can be viewed as biological standards that not only remain stable over time for a given donor but are associated with a rather low inter-individual variation, at least in healthy individuals. This has been emphasized for CD4 on positive T lymphocytes at ~50,000 molecules/cell [23,46] and has been proposed for CD45 calibration on peripheral-blood mature lymphocytes [47], measured at ~200,000 molecules/cell [17]. These levels depend on the differentiation status, as observed on differentiating bone-marrow leucocytes [48] as well as on immature leukemic cells [49].

Similarly, most major platelet membrane receptors can be considered as blood-borne biological standards due to their relatively homogeneous expression levels on the surface of resting platelets from healthy individuals. It may be hypothesized that this low inter-individual variation could be linked to the often-vital biological functions associated with these membrane receptors. Thus, the definition of normal ranges is fully justified as well as the search for abnormal expression levels that may be linked to differentiation, aging, activation, genetic polymorphism, pathology, and environmental factors (including food and drugs) as well as therapeutic interventions [50]. Several examples are provided below to illustrate these general statements.

## 6. Aging

There are two concepts in the impact of age, one is that of the individual/patient and the other one is that of platelets that age over about 11 days after production by megakaryocyte fragmentation.

Hézard et al. [51] have evaluated expression levels for the major platelet receptors in neonates, infants, children and adults, both in quiescent state and after full activation with TRAP, using absolute quantitation. Significant differences were observed in the basal levels of GPIIb/IIIa using a CD61 mAb, with median levels over 55,000 molecules per platelet in adults versus below 45,000 molecules in pediatric samples. This could not simply be due to differences in platelet size, although the low mean volume in neonates (6.8 fL) is different from that of both adults (7.9 fL) and children of various ages (7.7–7.9 fL), as other receptors behave differently. Surprisingly, all pediatric platelets, except those from neonates, could reach the same level as that measured on adult platelets upon TRAP activation, e.g., >70,000 molecules/platelet. Differences were also measured regarding the expression levels of CD62P after TRAP activation of ~6000 molecules per platelet in neonates up to a median close to ~12,000 molecules per platelet in adults.

Regarding platelet age, MHC Class I molecules (HLA-I) are one of the surface antigens to be quantified on platelets using radio-binding assays in early studies. These experiments suggested that the expression level of HLA-I could be an indicator of platelet age, with younger platelets showing the highest level of expression [52,53]. In a series of 12 individuals, the mean number of HLA Class I molecules per platelet was 81,500 ± 20,000 (range 54,800–116,200). Angénieux et al. [54] more recently normalized IF data to the size-related forward scatter parameter, a technological means to get rid of the impact of size and approach real density, which ideally would be expressed as molecules per µm^2^ of platelet surface [55]. Using the same anti-HLA-I mAb W6-32 and FCM analysis correlated to either thiazole orange staining, a marker of the “immature platelet fraction (IPF)”, or the intra-cytoplasmic “ribosomal P-antigen”, a feature of young platelets, these authors showed that the density of MHC Class I molecules (HLA-I) at the surface of platelets is a reliable marker to identify young platelets.

Finally, Veninga et al. [56] have recently shown that high GPVI expression is a feature of highly reactive juvenile platelets, which are predominantly found among the population of large platelets.

## 7. Genetic Polymorphisms and Phenotypic Variations

Several platelet membrane receptors show gene-related variations in their expression levels.

GPIa/IIa, a receptor for collagen, displays values that may differ by as much as one log between individuals, indicating variable capacities of collagen binding. These differences have been shown to relate to the C807T polymorphism of GPIa. The T807/A873 allele of the *GPIA* gene is an independent risk factor for the residual platelet reactivity on dual antiplatelet treatment, and it was shown, in a large population with acute coronary syndrome, that this allele is associated with higher platelet reactivity [57]. The TT genotype of the *GPIA* C807T polymorphism is associated with an increased susceptibility to ischemic stroke [58,59] or retinal vein occlusion [60]. However, it has no significant influence on the major adverse events occurring after coronary artery stenting [61] and does not associate with bleeding risk after percutaneous coronary intervention [62].

CD32 (FcγRIIa) carries a G > A polymorphism in the *FCRG2A* gene, leading to the replacement of a histidine (H) by an arginine (R) in position 131 of the extra-cellular domain. Depending on the two alleles, three phenotypes can be present, i.e., HR, HH or RR, respectively. Based on the differential binding affinity of two different mAbs against FcγRIIa [63], a quantitative phenotype can rapidly be determined that is correlated with the genetic polymorphism. The PLATELET FcγRIIa^®^ kit (Biocytex. RUO) contains two mAbs, one with a low affinity for the H131 allele and another that binds FcγRIIa independently of the polymorphism. The ratio of sABCs obtained with the two mAbs, ranging from >>1 for HH to <<0. 5 for RR and ~1 for HR, readily identifies the polymorphism. Another assay (CELLQUANT FcγRIIa^®^, Biocytex. RUO) measures the level of FcγRIIa on granulocytes using EDTA (and not citrate) anti-coagulated blood (Table 5).

The polymorphism influences the opsonizing capacity of neutrophils [64] and has implications for platelet activation [65]. It was reported to be a significant risk factor for anti-phospholipid syndrome (APS) [66] and systemic lupus erythematosus (SLE) in global [67] and Asian populations [68]. The FcγRIIA 131RR genotype was also reported to increase the risk of thrombosis in patients with heparin-induced thrombocytopenia [69].

Human Platelet Antigen (HPA-1) is the major allotypic Ag found on platelet GPIIIa and constitutes a target for alloimmunization. At least 16 polymorphisms have been identified on the HPA system [70,71]. Anti-HPA alloantibodies are responsible for fetal and neonatal alloimmune thrombocytopenia (FNAIT) or neonatal alloimmune thrombocytopenia (NAIT) [72], post-transfusion purpura (PTP) and platelet transfusion refractoriness (PTR). In Caucasians, the HPA-1a antigen is the most commonly involved. This is due to a variation (T176C) in the *ITGB3* gene leading to the L33P substitution in GPIIIa. In the Biocytex PLT HPA1 kit^®^ (CE), two mAbs have, respectively, a low affinity for the HPA-1b allele [73] or bind GPIIIa independently of the HPA-1 polymorphism, again allowing for a fast identification of the phenotype. Regarding functional and/or clinical implications, the HPA-1b phenotype has been reported to be associated with increased fibrinogen binding and platelet aggregation, potentially increasing the risk of myocardial infarction. It may also impact the severity of GT [74].

PAR-1 (proteinase-activated receptor 1), the main thrombin receptor on endothelial cells and platelets, plays a key role in platelet activation. Dupont et al. [27] evaluated the impact of *PAR-1* gene polymorphisms on the expression and function of PAR-1 on platelets using a Biocytex Platelet Calibrator kit^®^ (RUO) with two different mAbs assessing total PAR-1 and uncleaved PAR-1 expression. *IVSn* 14 A/T intronic variation was found to impact in vitro platelet activation, notably with the thrombin receptor activation peptide SFLLRN.

GPVI [75,76,77,78] is one of the platelet collagen receptors, responsible for platelet activation. Best et al. [79] measured a low density of GPVI as 3730 ± 453 ABC per platelet, with a limited effect of *GPVI* gene variations on GPVI expression levels, as confirmed by Joutsi-Korhonen et al. [80], who also observed an association of lower levels with myeloproliferative disorders. Since functional GPVI is associated with the formation of dimers on the platelet surface, the amount of dimers also requires quantitation. The Platelet Calibrator kit^®^ (Biocytex) technology has been applied and even refined for such sophisticated QFCM studies by Jung et al. [12], indicating that GPVI dimers account for ~30% of total GPVI on resting platelets and illustrating the effect of various agonists on this proportion. For example, thrombin increases this fraction to almost 50%, while the level of total GPVI remains globally unchanged at about 6000 molecules per platelet.

Bender et al. [80] highlighted that therapeutic interventions modulating the expression of GPVI and CLEC-2 may result in severely defective hemostasis and arterial thrombus formation in mouse models. Gitz et al. [81] showed that CLEC-2 and GPVI are expressed on CD41+ microvesicles, which would be predominantly derived from megakaryocytes in healthy donors, whereas microvesicles derived from activated platelets (and those from rheumatoid arthritis patients) express CLEC-2 only.

CD148, the only receptor-like protein tyrosine phosphatase, plays a role in platelet activation and is a positive regulator of hemostasis and arterial thrombosis in vivo [82]. The Biocytex Platelet Calibrator Kit^®^ was used to quantify CD148 on human platelets, which were found to express 2834 ± 90 molecules on their surface.

## 8. Monitoring of Therapy

In the first-in-human, randomized, placebo-controlled phase 1 study [83] that was conducted to evaluate the safety, tolerability, pharmacokinetics, and pharmacodynamics of the antiplatelet agent ACT017 (humanized Fab anti-GPVI, also known as glenzocimab) in healthy subjects, there was no change in platelet GPVI expression, as expected from pre-clinical studies. In this study, the level of GPVI expression at the platelet surface was assessed by direct IF using PE-coupled 1G5 (Biocytex. RUO), an mAb that binds to a different epitope than that recognized by ACT017. QFCM was not needed for monitoring since the stability of MFI was clear enough to show that ACT017 administration did not impact GPVI expression.

However, QFCM has been widely applied in other pre-clinical and clinical trials of therapeutic mAbs. Abciximab (anti-GPIIb/IIIa) in particular [84] has provided illustrations of the QFCM approach when the question was to define which level of receptor occupancy was needed to reach optimal therapeutic efficacy. The Biocytex Platelet GPIIb/IIIa occupancy^®^ kit (RUO) used the differential binding to GPIIIa of two reporter mAbs directed against different domains of the molecule, i.e., sensitive or insensitive to GP binding of the drug disintegrin, respectively. The latter thus measured total GPIIIa expression, while the former only accounted for the free receptor. Receptor occupancy was thus defined as the (total-free/total) ratio. The roles of mAbs used in such monitoring tests may differ depending on the drug tested, i.e., abciximab (ReoPro^®^) [85], eptifibatide (Integrilin^®^) [84] or tirofiban (Aggrastat^®^) [86]. This system has been widely used in pre-clinical and clinical studies when the question was to define the minimum receptor occupancy level required to provide ad hoc therapeutic efficacy. For example, Quinn et al. [85] reported that external receptor occupancy occurs within 3 min of an abciximab bolus and decreases over 15 days as abciximab disappears from the platelet surface. This receptor occupancy QFCM assay was also applied for subtle biophysical studies of modifications to the α2β3 integrin structure involved by such treatments.

## 9. Conclusions and Perspectives

As illustrated all throughout this review, the expression levels of molecules on almost any cell surface and especially platelet surface can be measured on an absolute basis, enabling reproducible evaluations, both over time and between instruments and laboratories, provided that (i) mAbs are available to recognize target molecules, (ii) staining is made under saturating conditions, (iii) all immunological tools (mAbs, secondary reagent, calibration beads) remain the same or have documented batch-to-batch consistency and (iv) the FCM instrumentation is properly maintained, as for all daily uses in laboratory medicine. This provides data expressed in sABC or mAb molecules per cell and possibly receptor copies per cell if the stoichiometry of mAb to Ag binding is known, most often corresponding to 1:1.

Besides descriptive studies providing copy numbers for known or newly discovered Ag/receptors, useful information can be derived from sABC measurements. Information includes (but is not limited to) correlations between platelet surface density and activation status, effect of aging, polymorphism dependence, genetic diseases, acquired disease states, and effect of a drug or an agonist. On an absolute basis, sABC values can provide reference normal (or sub-group-related) values enabling comparisons between healthy subjects, patients and treated patients.

The reference sABCs thus have potential applications as diagnostic, prognostic and treatment monitoring tools in a personalized medicine perspective, possibly in a fully automated artificial-intelligence-driven way in the future.

## Figures and Tables

**Table 1:** Classification of calibrators (“Multi-level, Type III, fluorescence Standards”) for quantitative flow cytometry (QFCM), according to Schwartz [13] and Poncelet [1].

Calibrators (Standards Type III)	Scheme	Features	Examples (Non-Exhaustive Lists)
**Type III A** **“Hard dyed” fluorescent beads** **Internal fluorochrome** *To check instrument linearity/sensitivity*	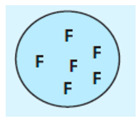	Long-term stability Robustness Sharp peaks Multiple colors Excited by various lasers NO spectral match with cell-bound fluorophores	Sphero-Rainbow^®^ Calibrator (Spherotech, Lake Forest, IL, USA) (RUO) CytoCal Multifluor (ThermoFischer, Waltham, MA, USA) (discontinued) Immuno-Brite^®^ multi-level beads (Beckman-Coulter, Brea, CA, USA) (CE-IVD)
**Type III B** **Surface fluorescent beads External fluorochrome** *For direct IF* *To calibrate bound fluophores per cell*	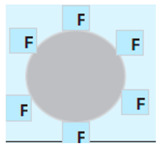	Limited shelf life Preferred cold storage (or lyophilized single-use) Spectrally matched with cell-bound fluorophores e.g., FITC, PE, APC, Pac Blue, Cy5 …	Quantum^®^ beads (C series) (Bang’s/ThermoFisher) (RUO) QuantiBrite^®^ (PE levels) (Becton-Dickinson, Erembodegem, Belgium) (RUO)
**Type III C** **Antibody-binding capacity beads** Non-fluorescent *For direct IF*	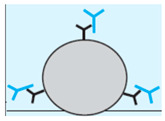	Non-fluorescent white polymer with surface-bound anti-mouse IgG (Fc) antibody Preferred cold storage mAb binding kinetics from cell-surface Ag	Quantum^®^ Simply Cellular beads (Bang’s/ThermoFisher) (RUO)
**Type III D** **IgG-coated beads** Non-fluorescent *For indirect IF*	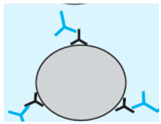	Non-fluorescent white polymer with surface-bound mouse IgG Preferred cold storage **Mimic stained cells** for indirect IF	QIFIkit^®^ (DAKO, BioCytex, Marseille, France) (RUO) CellQuant Calibrator, platelet calibrator, platelet kits e.g., PLT GP receptors (BioCytex) (RUO)
**Controls**			
**Stabilized cells**	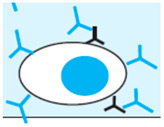	Stabilized cells with known membrane antigen densities	CytoTrol^®^; Immuno-Trol^®^ (CE-IVD); ClearLab Control Cells (Beckman-Coulter) (CE-IVD)

CE-IVD: European commission in vitro diagnostic; RUO: research use only.

**Table 2:** Platelet proteins possibly investigated in QFCM.

CD41/61(GPIIb/IIIa)	tetraspanins associated to platelet receptors or to microdomains in resting platelets
CD42 (GPIb/IX/V)	
CD31 (PECAM-1)	
CD36 (GPIV)	
CD49b/CD29 (GPIa/IIa)	
GPVI	
HLA-I	
CD34	
CD40L	
CD148	
CD109	
CLEC-2	
P-Selectin (CD62P)	
Tspan9 (NET-5)	
Tspan24 (CD151)	
Tspan30 (CD63	
Tspan32	
FcγRIIa (CD32)	
normal prion protein PrPc	

**Table 3:** Receptor density (sABC) values for the major platelet glycoproteins on quiescent versus activated (TRAP) platelets, from a healthy individual, a patient with alpha granules deficiency (gray platelet syndrome—GPS), a patient with biallelic Bernard–Soulier syndrome (BSS) each collected twice at various dates (personal data) and normal platelets (NPs) from multiple healthy donors (personal data).

	Healthy D1 *	Healthy D165 *	GPSD1	GPSD542	BSS D1	BSS D27	NPs **
**Quiescent state**							
GPIIb/IIIa (CD41) ***	68,795	65,491	50,584	49,114	100,074	98,111	51,000 ± 14,000
GPIIIa (CD61)	66,267	62,098	N.D.	N.D.	N.D.	N.D	N.D.
GPIb (CD42b)	41,295	48,980	35,161	35,106	7407	6791	38,000 ± 11,000
P-Selectin (CD62P)	<500	<500	<500	<500	<500	<500	<1000
**TRAP activation**							
GPIIb/IIIa (CD41)	128,291	158,458	78,554	73,819	106,904	114,515	85,000 ± 27,000
GPIIIa (CD61)	105,029	129,456	N.D.	N.D.	N.D.	N.D	N.D.
GPIb (CD42b)	21,122	39,281	23,217	21,477	5283	4634	19,000 ± 10,000
P-Selectin (CD62P)	12,935	13,673	1021	1134	4149	6621	>1000

* Results expressed as molecules/platelet. Data raised using CytoQuant GP, a previous version of PLT-GP receptors kit. ** Values determined internally on 40 samples drawn from normal adult donors (PLT-GP receptors kit insert data). *** As measured using an anti-complex reacting mAb. N.D.: not done.

**Table 4:** Receptor density (sABC) values for GPIIb/IIIa and GPIb on quiescent versus activated (TRAP) platelets in a familial study of Glanzmann thrombasthenia (personal data).

	Mother GT Heterozygous	Father GT Heterozygous	Daughter GT Homozygous	Normal Values **
Quiescent state				
GPIIb/IIIa (CD41/CD61)	31,532	30,386	995	51,000 ± 14,000
GPIb (CD42b)	42,749	35,830	54,120	38,000 ± 11,000
TRAP activation				
GPIIb/IIIa (CD41/CD61)	59,223	53,389	2642	85,000 ± 27,000
GPIb (CD42b)	33,086	23,274	29,665	19,000 ± 10,000

** Values determined internally on 40 samples drawn from normal adult donors (PLT-GP receptors kit insert data).

**Table 5:** Quantification of FcγRIIa using PLATELET FcγRIIa^®^ and CELLQUANT FcγRIIa^®^ kits provides phenotypic orientation about the H/R 131 polymorphism on platelets and granulocytes.

		PLATELET FcγRIIa			CELLQUANT FcγRIIa		
Polymorphism	ABC	Isotypic control	mAb1	mAb2	Isotypic control	mAb1	mAb2
H131/H131	mean	233	305	1384	979	2330	18,329
*n* = 16	SD	28	128	690	173	1003	2960
H131/R131	mean	250	807	855	920	14,103	15,104
*n* = 25	SD	34	306	281	152	3290	3190
R131/R131	mean	252	1747	848	1114	23,554	13,902
*n* = 16	SD	42	695	367	271	6065	3260

## Data Availability

No new data were created or analyzed in this study. Data sharing is not applicable to this article.

## Abbreviations

The following abbreviations are used in this manuscript:

ABC	Antibody-Binding Capacity
Ag	Antigen
BSS	Bernard–Soulier Syndrome
EV	Extra-cellular Vesicle
FCM	Flow Cytometry
FNAIT	Fetal and Neonatal AlloImmune Thrombocytopenia
GP	Glycoprotein
GPS	Gray Platelet Syndrome
GT	Glanzmann Thrombasthenia
IF	Immunofluorescence
mAb	Monoclonal Antibody
MEFL	Mean Equivalent Fluorochrome
MESF	Molecules of Equivalent Soluble Fluorochrome
MV	Microvesicle
NAIT	Neonatal AlloImmune Thrombocytopenia
PE	Phycoerythrin
PMT	Photo Multiplier Tubes
PTP	Post-Transfusion Purpura
PTR	Platelet Transfusion Refractoriness
QFCM	Quantitative Flow Cytometry
QIFI assay	Quantitative ImmunoFluorescence Indirect assay
QSC	Quantum Simply Cellular
sABC	specific ABC
TRAP	Thrombin Receptor Agonist Peptide
